# Influence of Multiple Infection and Relatedness on Virulence: Disease Dynamics in an Experimental Plant Population and Its Castrating Parasite

**DOI:** 10.1371/journal.pone.0098526

**Published:** 2014-06-03

**Authors:** Lorenza Buono, Manuela López-Villavicencio, Jacqui A. Shykoff, Alodie Snirc, Tatiana Giraud

**Affiliations:** 1 Ecologie, Systématique et Evolution, Université Paris-Sud, Orsay, France; 2 Ecologie, Systématique et Evolution, CNRS, Orsay, France; 3 Department Systématique et Evolution, Origine, Structure, Evolution de la Biodiversité, UMR 7205 CNRS-MNHN, Muséum National d'Histoire Naturelle, Paris, France; Virginia Tech, United States of America

## Abstract

The level of parasite virulence, *i.e.*, the decrease in host's fitness due to a pathogen, is expected to depend on several parameters, such as the type of the disease (*e.g.*, castrating or host-killing) and the prevalence of multiple infections. Although these parameters have been extensively studied theoretically, few empirical data are available to validate theoretical predictions. Using the anther smut castrating disease on *Silene latifolia* caused by *Microbotryum lychnidis-dioicae*, we studied the dynamics of multiple infections and of different components of virulence (host death, non-recovery and percentage of castrated stems) during the entire lifespan of the host in an experimental population. We monitored the number of fungal genotypes within plants and their relatedness across five years, using microsatellite markers, as well as the rates of recovery and host death in the population. The mean relatedness among genotypes within plants remained at a high level throughout the entire host lifespan despite the dynamics of the disease, with recurrent new infections. Recovery was lower for plants with multiple infections compared to plants infected by a single genotype. As expected for castrating parasites, *M. lychnidis-dioicae* did not increase host mortality. Mortality varied across years but was generally lower for plants that had been diseased the preceding year. This is one of the few studies to have empirically verified theoretical expectations for castrating parasites, and to show particularly i) that castrated hosts live longer, suggesting that parasites can redirect resources normally used in reproduction to increase host lifespan, lengthening their transmission phase, and ii) that multiple infections increase virulence, here in terms of non-recovery and host castration.

## Introduction

Parasites use host resources for their own reproduction and transmission, thereby reducing the fitness of their hosts, this reduction in host fitness being called virulence. The reduction in host fitness can result from decreased host lifespan and/or fecundity. The large class of castrating parasites mainly divert energy from host reproduction to parasite reproduction and transmission [1]–[3]. Most theoretical models predict that the optimal virulence for castrating parasites should be maximal, *i.e.*, total host castration that appropriates all host reproductive resources [4]–[7]. Indeed, each unit of resource allocated to host reproduction is lost for the parasite, while the parasite can gain from resource allocated to host survival if this increases its total fitness by lengthening the transmission phase. Castrating parasites are thus expected to have less negative impact on host survivorship than other types of parasites [2], [8] specifically appropriating resources allocated to host reproduction but sparing those allocated to host survival [9]. A few theoretical and experimental works even suggest that castrating parasites could be selected to divert supplemental resources away from host reproduction to host maintenance, leading to gigantism or increased survivorship [10]–[13]. Castrating parasitoids, in contrast, are not expected to be selected for extending host lifespan since by definition their free-living stage is only liberated at host death [1]–[3], [14]. Few experimental data are available so far to test theoretical predictions regarding the effects on host survivorship of castrating parasites that are transmitted throughout a host's life (*i.e.*, not parasitoids or other parasites only transmitted after host's death) [2], but see [15], [16].

Many theoretical studies have concluded that virulence should also be affected by the occurrence of multiple infections [17]–[20]. Infections of a host by multiple genotypes of the same parasite, *i.e.*, multiple infections, are extremely common in natural host-pathogen interactions [21] and lead to competition between co-infecting genotypes for limited host resources. Different kinds of competition may occur. One example, interference competition, or spite, arises when genotypes produce chemical antimicrobials that reduce or suppress the growth of other genotypes [22]–[26]. Another example is competition for resource uptake from the host. The intensity of competition between parasite genotypes sharing a host should depend on the relatedness between the different genotypes, with kin selection predicting that all types of competition should be reduced at high levels of relatedness to avoid damaging relatives [27].

Assessing the impact of castrating parasites on host mortality and understanding the effects of competition between different genotypes, in particular in terms of virulence, competitive exclusion and stable coexistence within hosts, requires following long-term dynamics of host-parasite interactions. Here, we studied the dynamics of the fungal pathogen *Microbotryum lychnidis-dioicae* (previously referred to as *M. violaceum*) in an experimental population of its dioecious caryophyllaceous host plant *Silene latifolia*. *Microbotryum lychnidis-dioicae* is a pollinator-borne parasite that castrates its host by destroying the ovaries of infected flowers and replacing pollen with fungal spores, which are dispersed by pollinators. In this dioecious host diseased flowers of female plants have aborted ovaries and both male and female flowers produce fungal spore-bearing anthers, without any pollen.

The *S. latifolia* - *M. lychnidis-dioicae* system is ideal for the study of virulence and of multiple infections for the following reasons. The disease becomes systemic so different parasite strains compete for space and for monopolising an infected plant [28]. Stems are produced every spring and die back in winter to a rosette stage, from which new stems are produced the following year. The fungus overwinters in the rosette and strains likely compete to gain access to meristems that will produce flowers in which the fungus sporulates and from which it then disperses [28]. Diseased flowers are sterile, so the main component of virulence for this castrating parasite can readily be estimated as percentage of castrated branches [29]. Other virulence components include mortality and lack of recovery, leading to permanent sterilization. Previous studies have found equivocal results of the effects of disease on *S. latifolia* mortality, with diseased plants suffering similar, less or more mortality in different years [30]–[33]. Numerous polymorphic markers are available [34]–[36] so we can differentiate between different fungal genotypes within plants, making it possible to monitor the fate of multiple infections over several years. Thus we know that multiple infections are common in natural populations, with different, related genotypes occupying different stems but never the same flower [37]. Experimental inoculations can also generate multiple infections [29], [32], [37]–[39]; sequential inoculation experiments reveal selective competitive exclusion: resident strains often exclude a challenging unrelated genotype while multiple infections are facilitated when genotypes are closely related [40].

In this study, we analyzed the dynamics of the anther smut disease by following an artificially inoculated population of *S. latifolia* throughout the entire host lifespan that lasted for a maximum of six years. We were particularly interested in detecting the effects of the castrating parasite and of multiple infections on two components of virulence, host non-recovery and host mortality. We also looked at the dynamics of relatedness of co-infecting strains within plants and at the degree of castration. For this goal, we generated an experimental population of ca. 1000 individuals of the caryophyllaceous plant *S. latifolia* infected with different genotypes of this fungal parasite. Plants were inoculated with different combinations of fungal genotypes differing in their degree of relatedness and additional natural transmission occurred in the garden. We have previously analyzed and published data from the first two years of this experiment, on the establishment of disease and patterns of multiple infections [29]. We reported that 1) strains that coexisted within plants were highly related, indeed far more related than the originally inoculated spore mixtures, and that less related fungal strains were more likely to be lost from multiple infections over the course of the first winter than were more closely related ones, suggesting exclusion of less related strains, 2) fungal strains in multiple infections restricted each others access to territory within the plant, but this restriction was less severe when strains were closely related, implying competitive exclusion that is moderated by high relatedness, and 3) per-flower spore production was greater from plants harboring multiple infections than from those with single infections, implying that multiple infections induced greater conversion of host resources to parasite propagules. Here we present data on annual disease dynamics, mortality and recovery from disease over the entire lifespan of the population. Indeed, though mortality was too low to be analyzed in the first two years of the experiment [29], all the plants had died within six years, allowing us to compare annual mortality between infected and healthy plants and those with single or multiple infections.

We thus report the dynamics of multiple infections within the experimental population across the entire five-year host population lifespan as well as the effect of the disease and of multiple infections on host mortality and recovery. Multiple infections were recognized here as the presence of different genotypes in different stems of a given plant and recovery was defined as the absence of symptoms (*i.e.*, no spores in any flowers of the whole plant) despite having produced spores the preceding year. Each year we assessed infection status, degree of castration of the plants and, using genetic markers, the number and identity of infecting genotypes per plant. This allowed us to estimate the relatedness between the fungal genotypes coexisting within plants. We compared the three main components of virulence, *i.e.*, the degree of castration, the rates of non-recovery and host mortality, between multiply infected, singly infected and healthy plants, and between sexes. We assessed disease spread in the population over time, degree of host castration and the dynamics of recovery and addressed the following questions: 1) Do the number of genotypes and degree of relatedness among coexisting genotypes within plants remain high over time? 2) Do the degree of host castration, non-recovery and host mortality, that are three important components of virulence, differ between single and multiple infections? 3) Is host lifespan affected by the disease?

## Materials and Methods

### Plant population, fungal strains and inoculations

To study the dynamics of disease and monitor the impact of the disease on a plant cohort, we created, in 2008, an experimental population of the host, *S. latifolia*, inoculated with strains of *M. lychnidis-dioicae*, in a garden of the University Paris Sud in Orsay (France). Seeds were collected from multiple mother plants in two populations from the same town (Orsay, France), one in 2005 and the other one in 2007. Only quantitative resistance is known in this system, with different plant families and populations showing different resistance levels in terms of probability of infection, *e.g.*, [41]–[43]. Given the relatively homogeneous origin of the seeds and the randomization of inoculations, there should be no effect of local adaptation influencing the results. Seeds were kept in a cold room until they were used. *Microbotryum* strains were collected near Orsay in 2001 for a metapopulation study [44]; later we collected strains from farther locations for studying the population structure across Europe [45], [46]. *Microbotryum* strains were kept dried in silica gel at 4°C until use. Seeds were sown on water agar in June 2008. The origin of fungal isolates used as inoculum is detailed in Table 1. In July 2008, 1021 individuals of *S. latifolia* were planted in the garden and individually tagged so that they could be identified across their entire lifespan. Plants were placed on a grid at 1 meter spacing from each of four closest neighbours and inoculated twice in July 2008, 15 days apart, with different mixtures of sporidia of *M. lychnidis-dioicae*. At this point, plants were relatively small with a few branches. For each fungal strain (diploid), we plated teliospores on GMB4 medium to multiply the products of meiosis [47]. Four inoculation treatments were applied (Table 2). Treatments A and B consisted of combinations of four diploid strains characterised using microsatellite markers. Strains in treatment A were genetically dissimilar, and therefore unrelated within the sampling universe of the experiment; those in treatment B were more similar and therefore related. For both treatments A and B there were four independent replicates of different strain combinations. A total of 94 plants were inoculated for each of the four strain combinations per treatment, yielding 376 plants for each of the treatments A and B. For the C treatment, a single diploid strain was inoculated on each plant. For this treatment, 13 different strains were used and 15 plants were inoculated with each of these single strains, yielding a total of 195 plants inoculated with a single strain each. Finally, 50 plants received no inoculation (treatment D). Inoculations were performed by adding a drop of fungal sporidia of both mating types for each strain suspended in tap water (approximately 10 ml of sporidia from each strain in 14 ml) at several points on all available meristems of the plants. Our objective was not to control for equal inoculum pressure between combinations or treatments or to compare virulence between treatments, but instead to obtain a high number of plants infected with multiple strains and to obtain a wide range of relatedness values between pairs of coinfecting strains, assessed *a posteriori*. Within mixtures of inoculated strains in treatments A and B, the relatedness, r, ranged from −0.1 to 0.2; relatedness was negative when genetic similarity among strains in the mixture was lower than expected by chance among strains drawn at random from the established genotypes in 2008. Our previous results showed that only highly related strains managed to co-colonize plants (with a mean of r = 0.5) [29]. Note that relatedness is relative: by definition, the mean relatedness among all fungal genotypes in the garden is zero. Relatedness higher than 0 within plants means that genotypes are more similar within plants than between random genotypes established in the garden the focal year. Higher relatedness of genotypes established within plants than in the inoculated mixtures results from a selective sampling of genetically similar genotypes within each infected plant, together with different genotypes succeeding among plants. No data were available on differences in virulence between the inoculated strains but little variation is known to exist in *M. lychnidis-dioicae* [48].

**Table 1:** Fungal inocula.

Strain ID	Place of collection	Year
3-04-01	Bonneville, near Orsay, France	2001
45	Brétonville, near Orsay, France	2001
45-01-07	Brétonville, near Orsay, France	2001
45-01-08	Brétonville, near Orsay, France	2001
45-03-07	Brétonville, near Orsay, France	2001
45-03-08	Brétonville, near Orsay, France	2001
45-06-02	Brétonville, near Orsay, France	2001
45-08-01	Brétonville, near Orsay, France	2001
45-08-04	Brétonville, near Orsay, France	2001
45-08-06	Brétonville, near Orsay, France	2001
45-09-15	Brétonville, near Orsay, France	2001
45-10-5	Brétonville, near Orsay, France	2001
49-06-02	Orsonville, near Orsay, France	2001
49-08-02	Orsonville, near Orsay, France	2001
49-08-04	Orsonville, near Orsay, France	2001
645	Toledo, Spain	2008
656	Brno, Czech Republic	2008
657	Vauchassis, France	2008
663-4	Bordeaux, France	2008
665-3	Mount Biokovo, Croatia	2008
670	Kecskemét, Hungary	2008
678	Baja, Hungary	2008
689	Ramegnies-Chin, Tournai, Belgium	2008
690	Ramegnies-Chin, Tournai, Belgium	2008
698	Tchernobyl, Ukraine	2008
7-21-04	Villebon, near Orsay, France	2001
7-21-05	Villebon, near Orsay, France	2001
7-24-06	Villebon, near Orsay, France	2001
7-24-09	Villebon, near Orsay, France	2001
SLAX	Mountain Lake Biological Station, Virginia, US	2008
SLBY4.1	Mountain Lake Biological Station, Virginia, US	2008

Strains of *Microbotryum lychnidis-dioicae* used for initial inoculations in the experimental garden of *Silene latifolia*.

**Table 2:** Inoculation treatments.

Treatment	Mixture	Strains			
A	1	665-3	45-01-07	654	663-4
A	2	45-06-02	656	678	689
A	3	SlAX	690	645	657
A	4	698	45-08-06	670	SLBY4.1
B	1	7-21-05	3-04-01	7-21-04	7-24-06
B	2	45	7-24-09	45-03-08	45-03-07
B	3	49-08-02	45-08-01	45-08-04	45-01-08
B	4	45-09-15	49-06-02	49-08-04	45-10-5
C	1	665-3			
C	2	689			
C	3	SlAX			
C	4	7-21-05			
C	5	49-08-02			
C	6	45			
C	7	645			
C	8	7-24-06			
C	9	45-03-07			
C	10	670			
C	11	45-01-08			
C	12	663-4			
C	13	3-04-01			

Combination of s trains of *Microbotryum lychnidis-dioicae* used for initial inoculations in the experimental garden of *Silene latifolia*.

Because artificial inoculations in this system often fail to cause disease, we knew our protocol would yield a garden with healthy plants in addition to the non-inoculated controls (internal controls) as well as diseased plants, and among the latter, plants with single infections and others with multiple infections, allowing comparison among healthy plants and diseased plants with single or multiple infections. We verified *a posteriori* that inoculated plants that had never expressed disease symptoms did not differ in mortality rates from those that had not been inoculated.

Numerous additional strains of *M. lychnidis-dioicae*, signalled by the appearance of novel alleles, also colonized the plants, presumably transmitted by pollinators in the garden [29]. Crosses between strains also probably occurred over the course of the experiments. The regular arrival of new fungal strains in the garden and crosses between inoculated and naturally transmitted strains as the source of new infections made it impossible to follow and compare success of individual fungal genotypes over the entire course of the experiment, and to follow individual strains over multiple years. We monitored, each year over the lifespan of each plant, the following traits: disease status, the number of different fungal genotypes coexisting within each plant and their relatedness (*i.e.*, their genetic similarity as compared to that expected by chance given allele frequencies in the garden that given year), the degree of castration (only after 2009, see [29]) and the death of plants. We recorded the data and sampled the strains once a year for each plant, in July, *i.e.*, during the summer peak of flowering. Data are therefore a single annual snapshot of the disease status for each plant.

### Sampling and genotyping

We collected one bud per infected branch once a year. We extracted DNA from one anther of each bud as previously described [44]. Six microsatellite markers were used: E14, E17, SL9, SL19, SL16 and SVG5 [34], [36] using the same protocol as previously described [44]. We did not genotype strains from the very few diseased plants in 2012.

### Genetic and statistical analyses

The relatedness between genotypes co-occurring in each plant was calculated using the software Relatedness based on the Queller model [49]. It estimates the proportion of shared alleles between individuals compared to the probability of sharing alleles by chance given the allele frequencies in the population. The allele frequencies used to estimate relatedness among fungal genotypes within plants in each year were those estimated in the garden in the focal year. This relatedness value is an estimation of the probability of sharing the same alleles at specific loci, for example those responsible for cooperation, which is the important parameter for evolution of altruism and is therefore used in theoretical models investigating the evolution of virulence.

Statistical analyses were performed using JMP, version 5 statistical package (SAS-institute). Logistic regressions were performed for analyzing the recovery and mortality for each plant as functions of year, plant sex, disease status (diseased versus healthy or single infection versus multiple infection), relatedness among co-infecting strains and/or the degree of castration. We fitted full models and simplified them by removing non-significant interactions and factors. We sequentially removed the non-significant main effects when they were not central to the questions being posed, *e.g*. plant sex, in order to increase our ability of detecting interesting main effects. In cases where removing some main effects did not improve explicative power of the model, we kept them in the model. “Year” was always kept as an explanatory variable in the analyses, because it functions as a blocking factor. Some factors could not be included together in the same model as they were not compatible (for instance “multiple infections versus single infections” and “relatedness among strains within plants with multiple infections”, or “diseased versus health plants” and “multiple versus single infections”); their effects had therefore to be tested in separate models. Multiple comparisons for means across years were performed with non-parametric Kruskal-Wallis tests. For the analysis of longevity, the number of years until death was recorded for each plant and an analysis of variance was performed to test whether plants that had been diseased for at least one year had a shorter or longer lifespan than those that had remained healthy throughout their lives. Because not all surviving plants flowered every year we have data only for 587 plants for this analysis, *i.e.*, those having flowered all years and for which we could therefore score the disease status all years. The data are available in the Table S1.

## Results

### Dynamics of plant mortality, recovery and new infections

**Figure 1:**
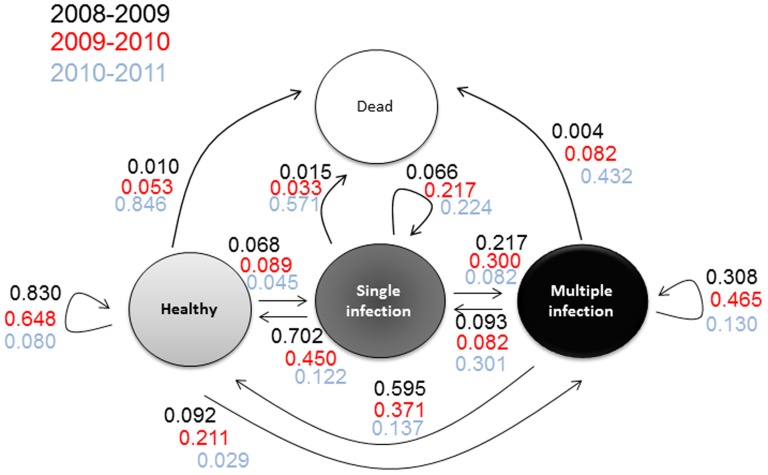
Dynamics of the *Silene latifolia* experimental population. Rates of new infections, recovery, acquisition of multiple infections and mortality are indicated, for the pairs of years for which data were available; 1021 seedlings were initially planted of which 95% were inoculated with *Microbotryum lychnidis-dioicae* strains. “Healthy” plants were those for which no symptoms could be observed, *i.e.*, no spores in the anthers of any flowers; “diseased” plants were those for which spores were observed in the anthers of at least some flowers; “single infection” refers to plants in which a single genotype was detected using microsatellite markers for genotyping the spores in the different diseased stems; “multiple infection” refers to plants in which at least two different genotypes were detected using microsatellite markers for genotyping the spores in the different diseased stems.

Figure 1 summarizes the transitions among healthy and singly and multiply infected plants within the experimental population from 2008–2011. Mortality was low between 2008 and 2010, with ca. 1% between 2008 and 2009 and 5% between 2009 and 2010. A peak of mortality occurred in the winter of 2010–2011, with the death of 69% of the 2010 population. Only 28 plants were then still alive in the summer of 2012, representing 2.7% of the initial population. All plants were dead in 2013. We have therefore followed the host cohort over its entire lifespan.

**Figure 2:**
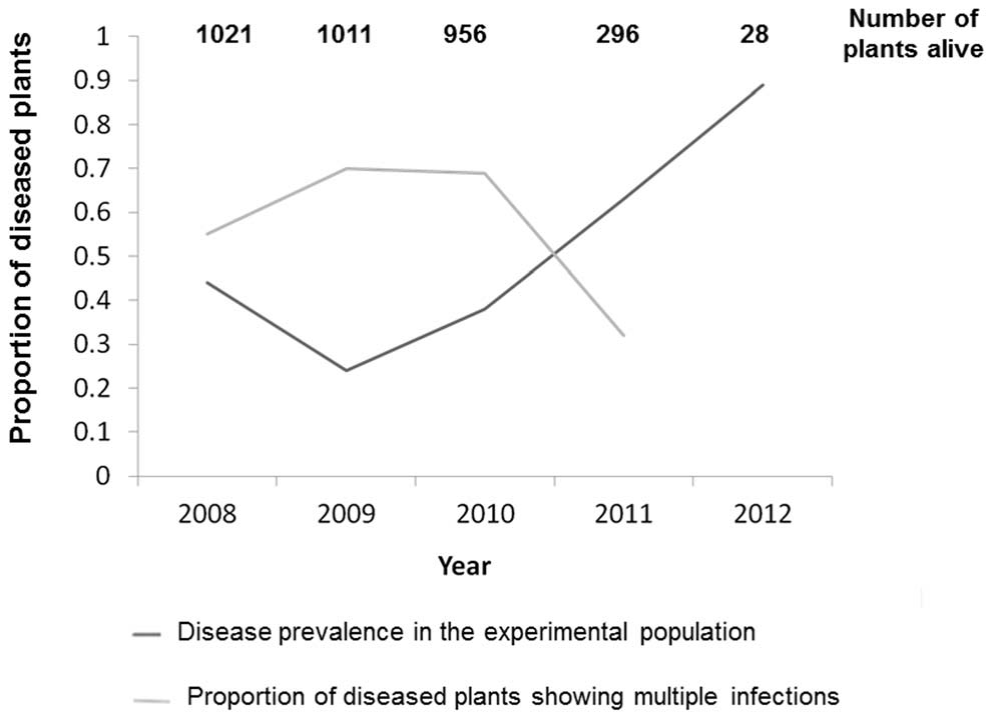
Dynamics of disease prevalence. Proportion of diseased plants, *i.e.*, with spores in the anthers of their flowers, dark grey, in the *Silene latifolia* experimental population across five years, with initially 1021 plants, 95% inoculated with *Microbotryum lychnidis-dioicae*; In light grey, proportion of diseased plants showing multiple infections across years, as assessed using microsatellite markers designed for *M. lychnidis-dioicae*; The number of plants still alive each year is indicated at the top.

The recovery rate (percentage of plants in a focal year that showed no symptoms, *i.e.*, no spores in any flowers, despite having produced spores the preceding year) was quite high in 2009 (64% of the plants that displayed diseased flowers in 2008 showed no symptoms in 2009), but decreased in subsequent years. Conversely, the rate of new infections, *i.e.*, of plants producing fungal spores while having shown no symptoms the preceding year, increased over time, from 16% in 2009 to 38% in 2011. Overall, the pathogen continued to colonize the population and to establish durable infections, reaching 89% prevalence in 2012 (Figure 2). The disease thus appeared dynamic and spreading across years (Figures 1 and 2).

**Figure 3:**
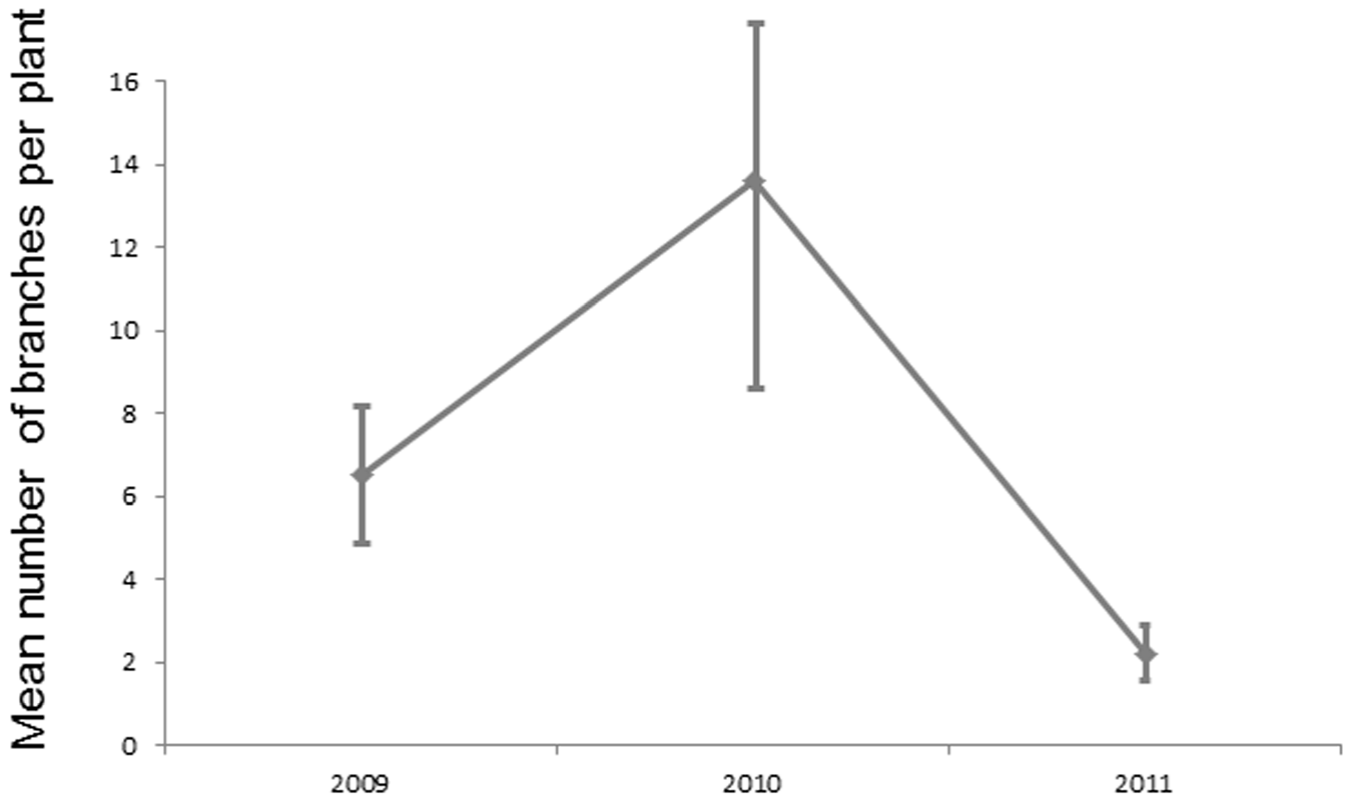
Dynamics of plant size. Mean number ± SD of branches per plant (healthy and diseased pooled) for each year where these data have been recorded.

**Figure 4:**
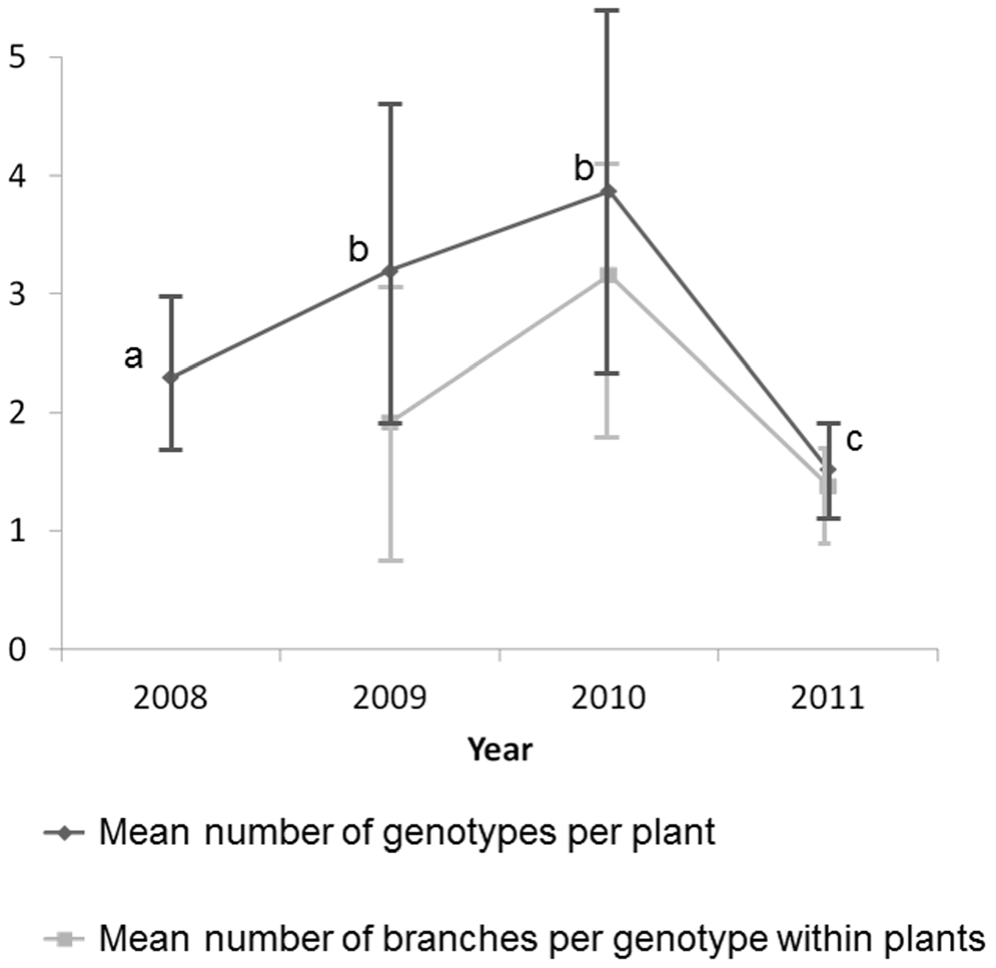
Dynamics of the number of fungal genotypes. Mean ± SD number of *Microbotryum lychnidis-dioicae* genotypes per *Silene latifolia* plant across years in the experimental population (dark grey) and mean ± SD number of branches occupied per genotype within plants (light grey).

**Figure 5:**
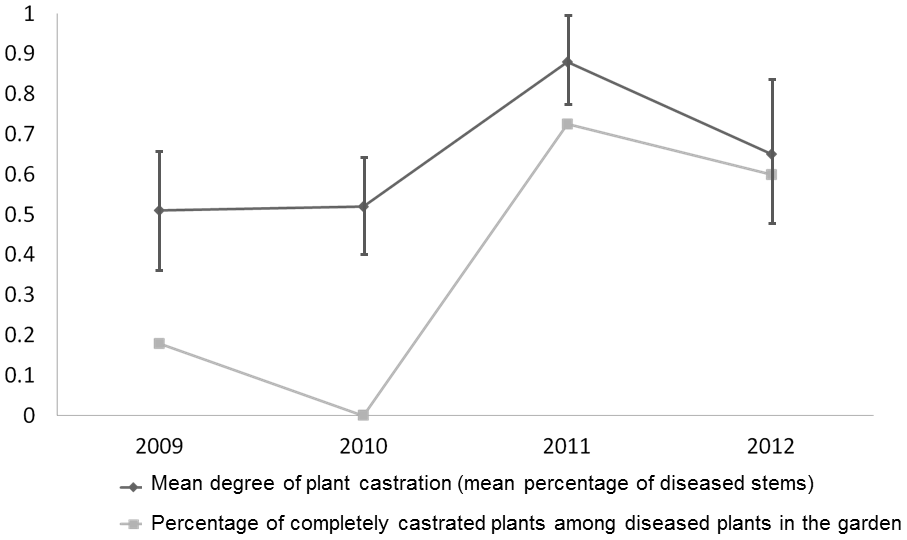
Dynamics of allele numbers. Allele numbers found in *Microbotryum lychnidis-dioicae* per microsatellite marker, in the inoculated strains and in the *Silene latifolia* experimental garden across years.

The percentage of plants infected by multiple genotypes increased from 55% of diseased plants in 2008 to 70% in 2009 and 2010 (Figure 2), because of accumulation of new infections and maybe also because of the increase in plant size (Figure 3). The percentage of plants infected by multiple genotypes then decreased to 32% of diseased plants in 2011 (Figure 2), not because of additional mortality of multiply infected plants, but probably because older plants became smaller (Figure 3). The mean number of genotypes per host showed the same pattern, with an increase from 2 to 4 between 2008 and 2010 and a decrease to 1–2 genotypes in 2011 (Figure 4). The mean number of alleles per marker in the garden also increased between 2008 and 2010, and then decreased in 2011 (Figure 5). New strains therefore regularly established in the garden.

**Figure 6:**
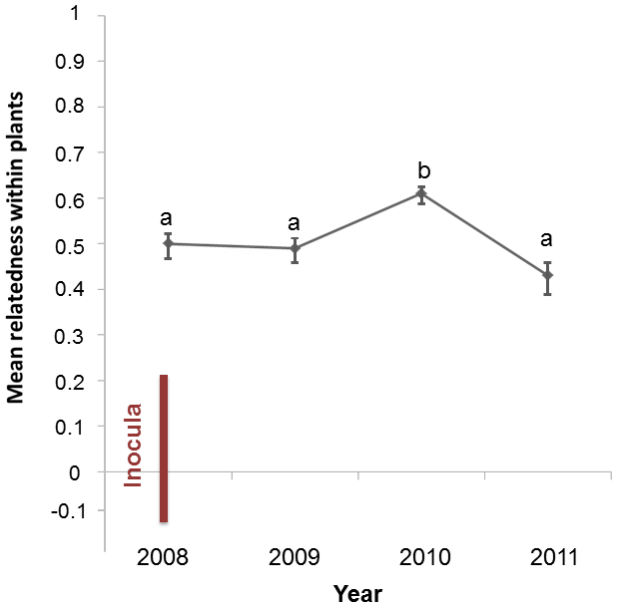
Dynamics of the degree of castration. Mean proportion of branches showing spores in the anthers of their flowers per diseased plant ± SD across year (dark grey) and percentage of completely castrated plants among diseased plants, *i.e.*, with all their flowers diseased, bearing spores (light grey).

The degree of castration, a component of parasite virulence, was measured as the proportion of castrated branches for each diseased plant. The mean degree of castration per plant remained stable at 50% in 2009 and 2010, increased sharply to 88% in 2011, and decreased to 65% in 2012 (Figure 6). Castration of diseased plants was not total for most plants in the first two years, in contrast to theoretical expectations for the optimal level of virulence for castrating parasites.

**Figure 7:**
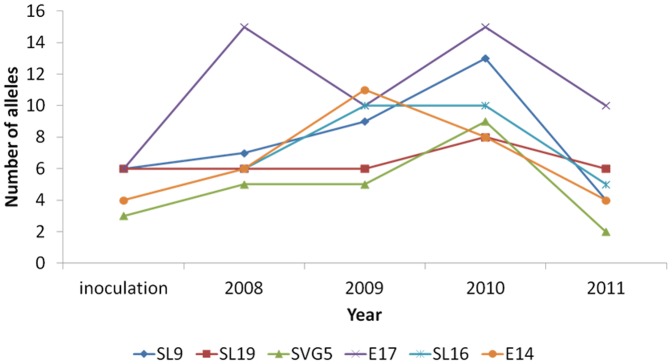
Dynamics of the relatedness within plants. Mean relatedness ± SD among *Microbotryum lychnidis-dioicae* genotypes co-occurring within plants across year; Relatedness within plants is the degree of similarity among fungal genotypes within a plant normalized by the degree of similarity of random genotypes in the garden; random expectation for relatedness is 0. The observed relatedness within plants is ca. that for full siblings (0.5). Means with the same letter are not significantly different, according to the Kruskal-Wallis test. The point in 2008 shows the relatedness among established genotypes within diseased plants; the bar represents the range in relatedness among genotypes inoculated together into the plants in the multiple infection Treatments A and B (relatedness was negative when less similar genotypes than a random sample were mixed).

The mean relatedness among genotypes within plants was higher than within any inoculum mixture, as shown and discussed previously [29], and remained at a high level throughout the entire host lifespan despite the dynamics of the disease, with recurrent new infections and recoveries (Figure 7). The mean value of relatedness among strains within plants remained around 0.5, the value of relatedness between diploid siblings, or even higher in 2010, while random expectations for relatedness is 0 and complete identity is 1 [49]. Relatedness among strains within plants thus remained high, with alleles significantly more similar within plants than expected by chance given the allele frequencies in the garden each year, as shown by the standard deviations not overlapping with 0 (Figure 7). This confirms that only highly related genotypes can co-exist within plants.

### Effect of the parasite on host longevity and parameters influencing virulence (non-recovery, mortality and castration)

**Figure 8:**
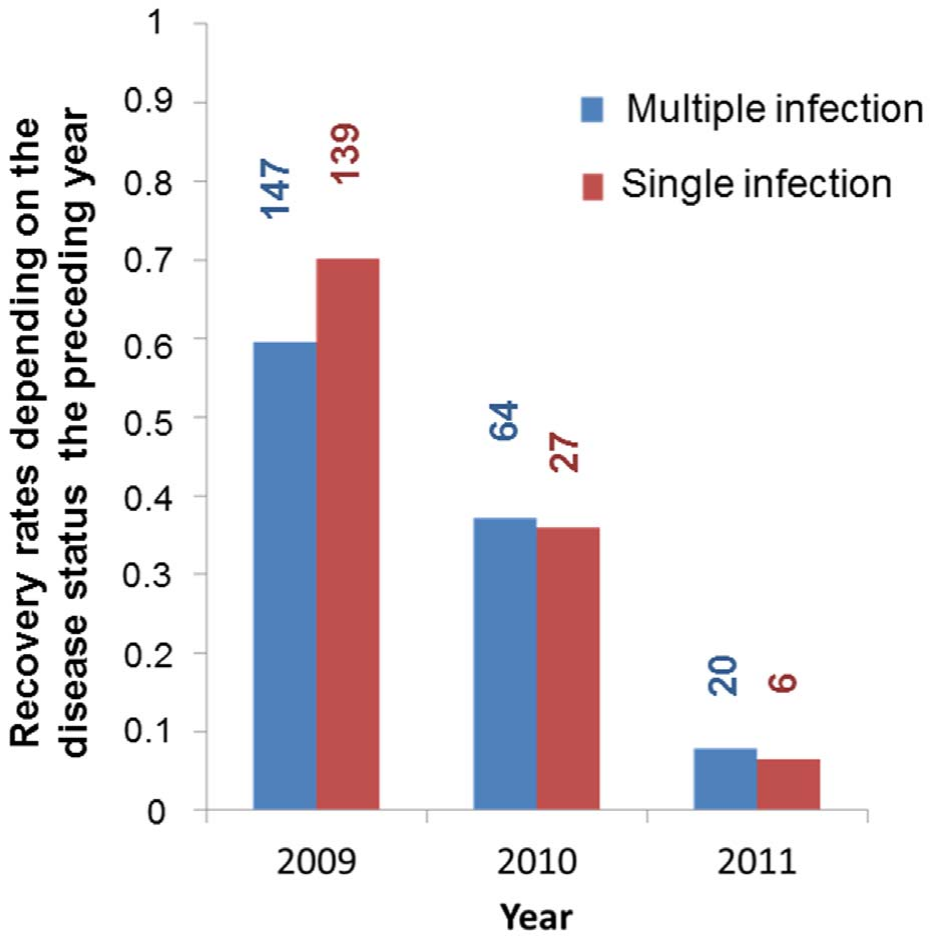
Plant recovery. Recovery rate per year in the *Silene latifolia* experimental garden depending on the plant infection status with *Microbotryum lychnidis-dioicae* (single versus multiple infections). The number of plants having recovered each year is indicated for each class. Recovery refers to absence of symptoms of a plant (*i.e.*, no spores in anthers in any of the flowers) that had been diseased the previous year, *i.e.*, with spores in anthers of at least some flowers. “Single infection” refers to plants in which a single genotype was detected using microsatellite markers for genotyping the spores in the different diseased stems; “multiple infection” refers to plants in which at least two different genotypes were detected using microsatellite markers for genotyping the spores in the different diseased stems.

Recovery was significantly less likely for plants that had been infected by several strains the preceding year than for plants infected by a single strain (Table 3, Figure 8). Multiple infections thus decreased the probability of recovery, increasing this component of virulence, though this effect appeared only obvious in 2009, after the first winter (Figure 8), the year of highest recovery rates. Recovery did not differ significantly between male and female host plants, nor was it influenced by relatedness among co-infecting strains or the degree of castration the preceding year (Table S2).

**Table 3:** Analysis of recovery rates.

	D.f.	χ^2^	p
**Year**	2	57.73	<0.00001
**Infection status (multi ** ***vs*** **. single infection) the preceding year**	1	5.55	0.018

Logistic regression of the recovery rate of *Silene latifolia* plants in the experimental garden as a function of year and infection status (multi *vs.* single infection by *Microbotryum lychnidis-dioicae*) the preceding year. The interaction between year and infection status was not significant. N = 766.

**Figure 9:**
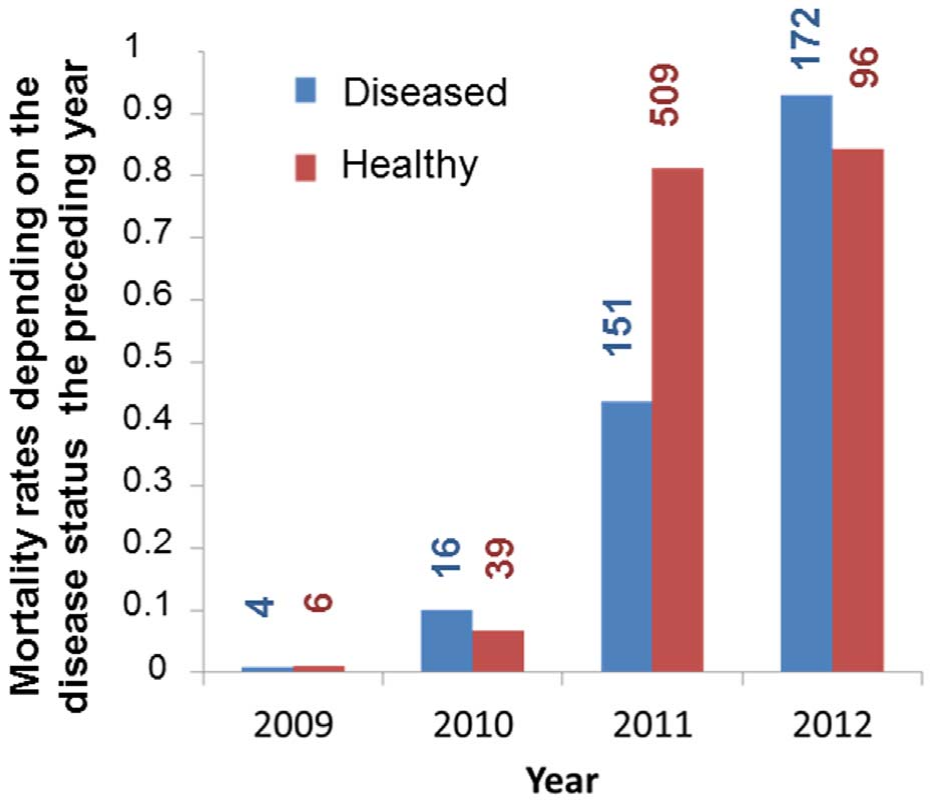
Plant mortality depending on disease status. Mortality rate in the *Silene latifolia* experimental garden across years and between plants differing in disease status with the anther smut *Microbotryum lychnidis-dioicae* the preceding year; mortality rate differed between diseased and healthy plants and among years.

**Figure 10:**
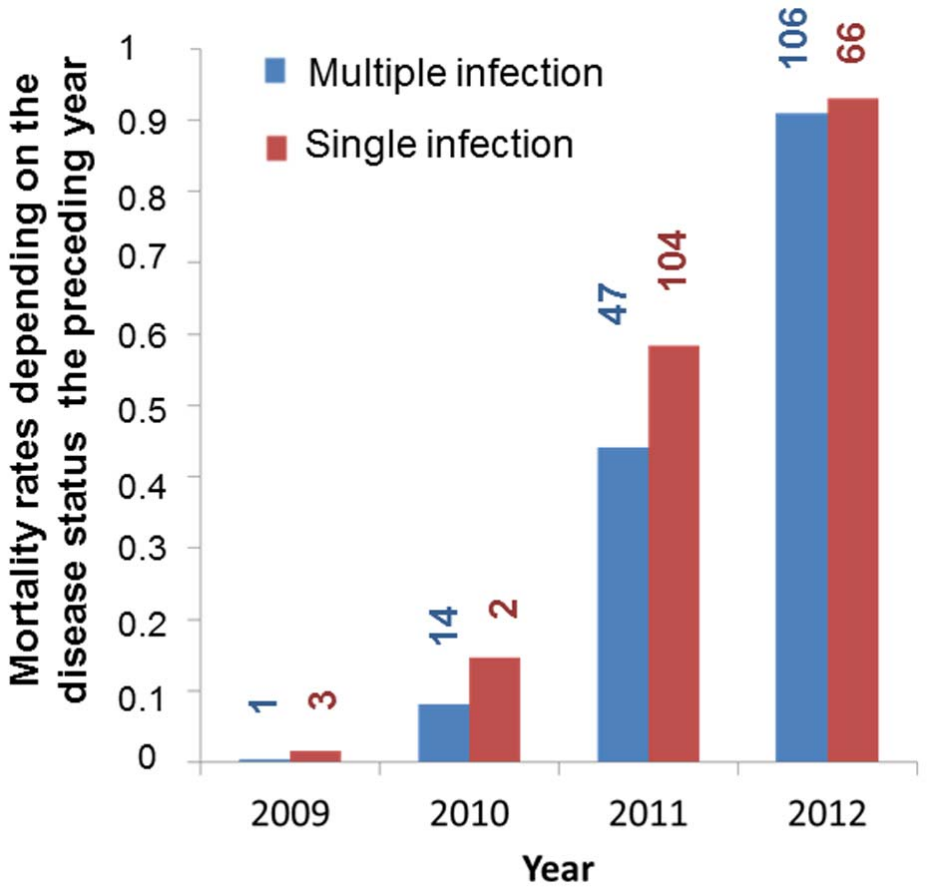
Plant mortality depending on multiple infections. Mortality rate depending on infection status the preceding year (multiple versus single infection); the mortality rate is not significantly different between plants with single versus multiple infections. “Single infection” refers to plants in which a single genotype was detected using microsatellite markers for genotyping the spores in the different diseased stems; “multiple infection” refers to plants in which at least two different genotypes were detected using microsatellite markers for genotyping the spores in the different diseased stems. The number of deaths each year is indicated for each class.

As expected for castrating parasites, *M. lychnidis-dioicae* did not increase host mortality. Mortality varied across years but was generally lower for plants that had been diseased the preceding year, though the strength of this effect appeared to vary across years (Table 4; Fig 9), appearing strongest in 2011, the year of the highest mortality rate before general death by senescence (Fig 9). Disease influenced longevity, with plants that had been diseased for at least one year living longer than those that had remained healthy throughout their lives (F_1, 585_ = 46.93, p<0.0001), by almost 5 months on average. We tested whether this could have resulted from confounding processes, for example, whether larger plants both lived longer and, because they attract more pollinators, were more likely to contract the disease. However the total number of stems, a measure of plant size, had no significant effect on plant mortality (Table S3), suggesting that the observation was not driven by plant-size effects. Mortality did not differ significantly between inoculation treatments, in particular between healthy non-inoculated and inoculated plants (Tables 4 and S3), confirming that healthy but inoculated plants in the garden constitute good controls for the effect of the disease on mortality, even though they had been challenged by the fungus. Mortality did not differ significantly between male and female host plants (Table S3). Multiple infections tended to increase the beneficial effect of the parasite on plant survivorship (Figure 10) but the effect was not significant (Table S3). As already found in the previous study on the first two years of the population, multiple infections increased the degree of plant castration, another component of virulence (Table 5).

**Table 4:** Analysis of mortality rates.

	D.f.	χ ^2^	p
**Year**	2	532.67	<0.00001
**Disease status (healthy ** ***vs*** ** diseased) the preceding year**	1	5.42	0.0199
**Interaction between year and disease status**	2	33.55	<0.00001
**Inoculation treatment**		3.55	0.3138

Logistic regression of mortality rate of *Silene latifolia* plants in the experimental garden as a function of year, disease status (plants without any anther smut disease symptoms, *vs* those showing spores in at least some flowers) the preceding year, and inoculation treatment (non-inoculated plants, single strain inoculum, multiple related strains of inoculum, or multiple unrelated strains of inoculum). N = 2547.

**Table 5:** Analysis of the degree of castration.

	D.f.	Sum of squares	F ratio	p
**Year**	2	7.329	51.29	<0.00001
**Multiple infection**	1	2.794	39.10	<0.00001

ANOVA on the degree of castration of *Silene latifolia* plants in the experimental garden as a function of year and multiple infections (plants with a single versus multiple genotypes). N = 433.

## Discussion

In this study, we analyzed the dynamics of the disease for a castrating fungal parasite under conditions of single *vs.* multiple infections. We followed an artificially inoculated population of plants throughout the entire host lifespan. We were particularly interested in detecting the effects of the castrating parasite and of multiple infections on important components of virulence that could not be analyzed in the study on the two first years of the experimental garden [29], *i.e.*, host non-recovery and host mortality. We also looked at the dynamics of relatedness of co-infecting strains within plants and at the degree of castration over the entire duration of the population, both of which had already been analyzed in the two first years of the garden [29].

### Prevalence and dynamic of the disease

The overall prevalence of the disease increased over time; four years after the beginning of this study most of the plants were diseased. The rates of multiple infections were high, as observed in previous experimental and natural populations [32], [37]–[40]. We were also able to observe the dynamics of the number of genotypes per plant, their degree of relatedness, their degree of castration of the host and rates of recovery and death in the population. The disease appeared dynamic, both in terms of infection status per plant and of number of fungal genotypes per plant. This highlights the need of experimental population studies across several years for a proper understanding of disease ecologies.

### Recovery

We cannot know whether the fungus was still present within plants that we considered recovered, *i.e.*, those that expressed no symptoms (spore production) the year following an occurrence of symptoms. However, even if recovery did not involve complete elimination of the fungus, *i.e.*, even if *M. lychnidis-dioicae* maintained symptomless infections, such cryptic infections allowed the plant to produce pollen and seeds and therefore to gain non-null fitness, greatly reducing virulence, which is what we wanted to measure.

The recovery rate was quite high the first years but decreased over time, remaining, however, non-negligible, as in previous population surveys [30], [31], [33], [50]. Interestingly, plants infected with multiple pathogen strains were less likely to recover than plants infected by a single strain. Thus, multiple infections decreased the probability of recovery, leading to greater disease-induced reduction in fitness. This suggests that multiple infections are more difficult to combat and clear. The lower recovery rate of plants with multiple infections is unlikely to be an indirect effect of the number of stems affected by the disease because, although multiple infections overall castrate a higher proportion of available stems [29], percentage castration did not significantly influence recovery probability. Similarly, we found no significant role of relatedness among genotypes on recovery, suggesting that the poorer recovery of multi-infected plants was not related to possible mechanisms of cooperation among related genotypes to fight the host defence machinery [51]. In plants, defence against pathogens can be genotype-specific, *i.e.*, more efficient against certain pathogen genotypes [52], [53]. The tendency of lower recovery of plants with multiple infections may be due to genotype-specific resistance, as it suggests that the plant defence is not similarly efficient against all genotypes. Though all-or-nothing gene-for-gene-resistance *sensu* [52] is not known for *Microbotryum*, plant genotypes vary in their probability of becoming diseased depending on the parasite genotype they confront, with variation both among and within host and parasite populations [41], [48]. For inoculation of the experimental population, we used *M. lychnidis-dioicae* strains from several geographic origins, so genotype-specific resistance is likely in our experiment. It should be noted that the higher recovery under single infection was evident only after the first winter, which was also the year of the highest recovery rates. The following years, the disease may have been better established in plants and/or environmental conditions may have been more favourable to the fungus. This highlights the importance of following cohorts over their entire lifespan for integrating fitness effects across years for ecological studies.

### Relatedness among coinfecting strains

The number of genotypes per plant increased over the first three years and their relatedness remained high, indicating that only significantly related newly colonizing genotypes were capable of colonizing and coexisting in the same host individual, confirming observations from experimental inoculations [39], [40], natural populations [37] and from the study of the two first years of the garden [29]. This cannot be due to specific adaptation of fungal genotypes to these plants because relatedness is relative: fungal genotypes coexisting within plants were genetically more similar than expected if all genotypes established in the garden had parasitized plants at random. All plants were grown from seeds collected from two neighbouring populations from Orsay, representing a homogeneous genetic background since plant populations are not strongly genetically differentiated [54]. The high relatedness within plants therefore cannot result from local adaptation to Orsay plant genotypes. Furthermore, this fungus is not locally adapted to its host populations [43]. The high relatedness within plants is thus far more likely to result from non-kin exclusion.

In the last two years of the garden, as plants became more castrated, the number of genotypes per plant decreased. This suggests that, in plants saturated by *Microbotryum*, resident strains could exclude new challengers and even displace some resident genotypes, as all available space within the plant became occupied. Such exclusion of competitors has been reported in some animal pathogens, such as trematodes in snails [55], [56]. The decrease in the number of genotypes per plant and of overall numbers of alleles in the garden is also related to plant senescence, as the number of stems per plant decreased in the last two years.

Contrary to expectations for a castrating parasite, not all plants appeared completely castrated, even after years of disease. In the two last years, still 30–40% of diseased plants were not completely castrated. This may be due to suboptimal adaptation of the parasite to these hosts or rather to the ability of plants to prevent complete systemic invasion. In particular, plants may be able to redirect flower production to new, not yet infected meristems, as suggested in a *Cordia nodosa* plant castrated by *Allomerus* parasite ants [57]. Individual stems however cannot be followed from one year to another in *S. latifolia* to test for this effect, because stems die back in winter.

### Host castration

In accordance with the theoretical expectation of increase in virulence under multiple infections [17]–[20], the degree of host castration increased under multiple infections, as found and extensively discussed in the previous study on the first two years of the garden [29]. This fits with the expectation that parasites respond to the presence of competitor within their host by increasing their virulence to enhance their short-term transmission, possibly jeopardizing longer term transmission, by uptaking resources at a higher rate, to monopolize the resources before the competitors.

### Mortality

All the plants had died by the end of the experiment. *Silene latifolia* is known to be a short-lived perennial unlike some other Caryophyllaceae such as *S. acaulis* [58]. Mortality thus appeared to be the natural result of senescence, as we noted no extrinsic cause for plant death in the garden. Interestingly, we found an overall significantly increased lifespan for diseased plants. Theory suggests that castrating parasites divert host reproductive resources to the production of parasitic transmission structures, without necessarily reducing host lifespan, which would shorten their transmission phase [8]. A model even showed that castrating parasites could be selected for diverting resources to enhanced host maintenance and therefore possibly survivorship [10]. Our case therefore seems to meet theoretical expectations. *Microbotryum* indeed appears to have no detrimental effect on its host survivorship; on the contrary, infected plants lived longer, suggesting that the parasite, despite using host reproductive resources for its own transmission stages, liberates some to be used for host maintenance. The difference in mortality between diseased and healthy plants appeared strongest in 2011, which makes sense, as this was the year with the highest mortality rates, and therefore highest statistical power, aside from the last year where death was likely mainly due to senescence. This again highlights the need to follow populations across several years for making reliable ecological inferences on fitness. Our results suggested that multiple infections increased the beneficial effect of the parasite on plant survivorship although the effect was not significant. In a previous experimental population [32], multiple infections also increased survivorship probability, and significantly so.

The present study is to the best of our knowledge the first one experimentally showing that the effect of castrating parasites can lead to increased hosts' lifespan, although previous circumstantial evidence suggested this effect in several animal-parasite systems [6], [9], [13], [15], [16]. This may simply be that parasite reproduction and transmission is somehow less costly than plant reproduction or it may result from an active manipulation of the host, *i.e.*, being an extended phenotype of the parasite [59] that provides a longer transmission phase. Increased lifespan associated with castration (of non-parasitic origin) has indeed been observed in many species of animals (including humans) and plants [60]–[62]. Increased host lifespan may also result from a host strategy whereby longer life would allow reproduction in case of later recovery. It may even be that such host compensatory mechanisms are used by the parasites for its own benefit of increasing its transmission phase [63].

Interestingly we found no difference in mortality between male and female plants, either as main factor or in interaction with infection status, even though male and female reproductive structures are quite different. Indeed, fruits and seeds of female *S. latifolia* are probably individually more costly [64], though males produce more flowers. The finding that castrated female plants do not display lower mortality rates, despite probably saving more energy, points to a hypothesis of parasite manipulation rather than simple resource re-allocation. Similarly, there was no significant impact of the degree of castration on host survivorship, while the hypothesis of mere plant resource re-allocation would predict that plants with higher degrees of castration would save more energy normally allocated to reproduction and thereby live longer than plants with lower rates of castration. Indeed *M. lychnidis-dioicae* induces higher flower production in diseased plants [65], which could counter the advantage of the reduction of per flower/fruit cost of the sterile flowers.

One could imagine that the lower mortality of diseased plants could be the result of an artefact, if larger plants were less likely to die but at the same time also attract more pollinators, and thereby were more likely both to become diseased and to harbour multiple infections. This hypothesis however is not consistent with the non-significant effect of total number of stems on plant mortality.

Our findings of lower mortality of diseased *S. latifolia* plants contradict some results of previous studies on populations of this host-parasite system. Indeed, higher mortality has been reported among diseased plants than among healthy ones, although the difference was not consistently significant across years in these studies [30], [31], [33]. Though we found an overall average reduction in mortality for diseased plants this was only obvious in 2011, the other years showing very similar mortality of healthy and diseased plants. Thus data from a single or two years may be insufficient to reveal the influence of the disease.

### Conclusion

In this study, we used a fungal pathogen model to follow the dynamics of a castrating disease and of multiple infections during the entire host lifespan of an experimental host cohort. We were able to address important aspects in the study of virulence for parasites that has rarely been studied experimentally: we showed, as expected based on theoretical models, an overall increase of virulence under multiple infections, in terms of plant non-recovery and degree of castration, and an increased lifespan of host plants castrated by the parasite. The extended phenotypes of castrating parasites may be to redirect resources from host reproduction to host maintenance, thereby increasing the lifespan of the host and maximizing parasite's transmission. This has important consequences for our understanding of disease evolution, which can also have applied consequences for disease management. Indeed, if individuals suffering from a castrating disease live longer, this will affect the degree of disease transmission in the population. Studying the proximal reasons for extended lifespan in castrated individuals may also grant new insights into the proximal mechanisms of senescence. The effects of the disease on mortality and the impact of multiple infections on recovery could be detected only through the monitoring of the cohort over its entire lifespan as these effects were strong on a single year each. This further highlights the value of long-term studies, as selection does act on the integration of the different components of fitness across the entire lifespan.

## Supporting Information

Table S1 **Data file.**
(XLSX)

Table S2 **Additional analysis of recovery rates.** Logistic regression of the recovery rate of *Silene latifolia* plants in the experimental garden as a function of year, plant sex, relatedness among strains in plants with multiple infections the preceding year and percentage of castrated stems the preceding year; N = 103. This model is presented separately from the one in Table 2 because relatedness within plants in multiple infections cannot be tested in the same model as disease status (multiple versus single infection) because the “single infection” class does not have any relatedness data. The factors plant sex is non-significant when included in the model in Table 2 and it reduces the power to detect the significance of the other factors.
(DOCX)

Table S3 **Additional analysis of mortality rates.** Logistic regression of mortality rate of *Silene latifolia* plants in the experimental garden as a function of year, plant sex, disease status (multiple vs single infection) the preceding year, and the percentage of diseased stems the preceding year; N = 173. This model is presented separately from the one in Table 3 because the factor “single vs. multiple infections” cannot be tested in the same model as “diseased vs. healthy”. The factor plant sex is non-significant when included in the model in Table 3 and it reduces the power to detect the significance of the other factors.
(DOCX)

## References

[1] Jensen KH, Little T, Skorping A, Ebert D (2006) Empirical support for optimal virulence in a castrating parasite. PLoS Biol 4: e197. 16719563 10.1371/journal.pbio.0040197 PMC1470460

[2] Lafferty K, Kuris A (2009) Parasitic castration: the evolution and ecology of body snatchers. Trends Parasitol 25: 564–572. 19800291 10.1016/j.pt.2009.09.003

[3] Mageroy J, Grepperud E, Jensen K (2011) Who benefits from reduced reproduction in parasitized hosts? An experimental test using the *Pasteuria ramosa-Daphnia magna* system. Parasitology 138: 1910–1915. 21854675 10.1017/S0031182011001302

[4] Best A, White A, Boots M (2010) Resistance is futile but tolerance can explain why parasites do not always castrate their hosts. Evolution 64: 348–357. 19686267 10.1111/j.1558-5646.2009.00819.x

[5] Jaenike J (1996) Suboptimal virulence in an insect-parasitic nematode. Evolution 50: 2241–2247. 28565686 10.1111/j.1558-5646.1996.tb03613.x

[6] Obreski S (2005) Parasite reproductive strategy and evolution of castration of hosts by parasites. Science 188: 1314–1316. 10.1126/science.1145198 1145198

[7] O'Keefe KJ, Antonovics J (2002) Playing by different rules: the evolution of virulence in sterilizing pathogens. Am Nat 159: 597–605. 18707384 10.1086/339990

[8] Antonovics J (2005) Plant venereal diseases: insights from a messy metaphor. New Phytol 165: 71–80. 15720622 10.1111/j.1469-8137.2004.01215.x

[9] Baudoin (1975) Host castration as a parasitic strategy. Evolution 29: 335–352. 28555867 10.1111/j.1558-5646.1975.tb00213.x

[10] Bonds MH (2006) Host life-history strategy explains pathogen-induced sterility. Am Nat 168: 281–293. 16947104 10.1086/506922

[11] Ebert D, Carius HJ, Little T, Decaestecker E (2004) The evolution of virulence when parasites cause host castration and gigantism. Am Nat 164: S19–S32. 15540139 10.1086/424606

[12] Hechinger RF (2010) Mortality affects adaptive allocation to growth and reproduction: field evidence from a guild of body snatchers. BMC Evol Biol 10: 136. 20459643 10.1186/1471-2148-10-136 PMC2887408

[13] Keeney D, Waters J, Poulin R (2006) Clonal diversity of the marine trematode Maritrema novaezealandensis within intermediate hosts: the molecular ecology of parasite life cycles. Mol Ecol 16: 431–439. 10.1111/j.1365-294X.2006.03143.x 17217355

[14] Ebert D, Weisser WW (1997) Optimal killing for obligate killers: the evolution of life histories and virulence of semelparous parasites. Proc R Soc Lond B 264: 985–991. 10.1098/rspb.1997.0136 PMC1688549 9263465

[15] Hechinger RF, Lafferty KD, Kuris AM (2008) Diversity increases biomass production for trematode parasites in snails. Proc Soc R Soc Lond B 275: 2707–2714. 10.1098/rspb.2008.0875 PMC2605822 18700204

[16] Burns CE, Goodwin BJ, Ostfeld RS (2005) A prescription for longer life? Bot fly parasitism of the white-footed mouse. Ecology 86: 753–761.

[17] Alizon S, Hurford A, Mideo N, Van Baalen M (2009) Virulence evolution and the trade-off hypothesis: history, current state of affairs and the future. J Evol Biol 22: 245–259. 19196383 10.1111/j.1420-9101.2008.01658.x

[18] Brown SP, Hochberg ME, Grenfell BT (2002) Does multiple infection select for raised virulence? Trends Microbiol 10: 401–405. 12217504 10.1016/s0966-842x(02)02413-7

[19] Frank SA (1996) Models of parasite virulence. Quart Rev Biol 71: 37–78. 8919665 10.1086/419267

[20] van Baalen M, Sabelis WS (1995) The dynamics of multiple infection and the evolution of virulence. Am Nat 146: 881–910.

[21] Read AF, Taylor LH (2001) The ecology of genetically diverse infections. Science 292: 1099–1102. 11352063 10.1126/science.1059410

[22] de Roode JC, Helinski MEH, Anwar MA, Read AF (2005) Dynamics of multiple infection and within-host competition in genetically diverse malaria infections. Am Nat 166: 531–542. 16224719 10.1086/491659

[23] de Roode JC, Pansini R, Cheesman SJ, Helinski MEH, Huijben S, et al (2005) Virulence and competitive ability in genetically diverse malaria infections. Proc Natl Acad Sci USA 102: 7624–7628. 15894623 10.1073/pnas.0500078102 PMC1140419

[24] Gardner A, West S, Buckling A (2004) Bacteriocins, spite and virulence. Proc R Soc Lond B 271: 1529. 10.1098/rspb.2004.2756 PMC1691756 15306326

[25] Massey RC, Buckling A, ffrench-Constant R (2004) Interference and parasite virulence. Proc Soc R London 271: 785–788. 10.1098/rspb.2004.2676 PMC1691666 15255095

[26] Ojosnegros S, Beerenwinkel N, Antal T, Nowak MA, Escarmis C, et al (2010) Competition-colonization dynamics in an RNA virus. Proc Natl Acad Sci USA 107: 2108–2112. 20080701 10.1073/pnas.0909787107 PMC2836666

[27] Buckling A, Brockhurst MA (2008) Kin selection and the evolution of virulence. Heredity 100: 484–488. 18212805 10.1038/sj.hdy.6801093

[28] Schäfer AM, Kemler M, Bauer R, Begerow D (2010) The illustrated life cycle of *Microbotryum* on the host plant *Silene latifolia* . Botany 88: 875–885.

[29] López-Villavicencio M, Courjol F, Gibson A, Hood M, Jonot O, et al (2011) Competition, cooperation among kin and virulence in multiple infections. Evolution 65: 1357–1366. 21121914 10.1111/j.1558-5646.2010.01207.x

[30] Alexander HM, Antonovics J (1988) Disease spread and population dynamics of anther-smut fungus of *Silene alba* caused by the fungus *Ustilago violacea* . J Ecol 76: 91–104.

[31] Alexander HM, Antonovics J (1995) Spread of anther-smut disease (*Ustilago violacea*) and character correlations in a genetically variable experimental population of *Silene alba* . J Ecol 83: 783–794.

[32] Hood ME (2003) Dynamics of multiple infection and within-host competition by the anther-smut pathogen. Am Nat 162: 122–133. 12856241 10.1086/375539

[33] Thrall PH, Jarosz AM (1994) Host-pathogen dynamics in experimental populations of *Silene alba* and *Ustilago violacea*. I. Ecological and genetic determinants of disease spread. J Ecol 82: 549–559.

[34] Bucheli E, Gautschi B, Shykoff JA (1998) Isolation and characterization of microsatellite loci in the anther smut fungus *Microbotryum violaceum* . Mol Ecol 7: 665–666. 9633108

[35] Giraud T, Fournier E, Vautrin D, Solignac M, Shykoff JA (2002) Isolation of 44 polymorphic microsatellite loci in three host races of the phytopathogenic fungus *Microbotryum violaceum* . Mol Ecol Notes 2: 142–146.

[36] Giraud T, Yockteng R, Marthey S, Chiapello H, Jonot O, et al (2008) Isolation of 60 polymorphic microsatellite loci in EST libraries of four sibling species of the phytopathogenic fungal complex *Microbotryum* . Mol Ecol Res 8: 387–392. 10.1111/j.1471-8286.2007.01967.x 21585800

[37] Lopez-Villavicencio M, Jonot O, Coantic A, Hood M, Enjalbert J, et al (2007) Multiple infections by the anther smut pathogen are frequent and involve related strains. PloS Pathog 3: e176. 18020704 10.1371/journal.ppat.0030176 PMC2077905

[38] Day AW (1980) Competition and distribution studies of genetically marked strains of *Ustilago violacea* in the same host plant. Bot Gaz 141: 313–320.

[39] Gold A, Giraud T, Hood M (2009) Within-host competitive exclusion among species of the anther smut pathogen. BMC Ecol 9: 11. 19422703 10.1186/1472-6785-9-11 PMC2688501

[40] Koskella B, Giraud T, Hood ME (2006) Pathogen relatedness affects the prevalence of within-host competition. Am Nat 168: 121–126. 16874619 10.1086/505770

[41] Cafuir L, Antonovics J, Hood ME (2007) Tissue culture and quantification of individual-level resistance to anther-smut disease in *Silene vulgaris* . Int J Plant Sci 168: 415–419.

[42] Chung E, Petit E, Antonovics J, Pedersen A, Hood ME (2012) Variation in resistance to multiple pathogen species: anther smuts of *Silene uniflora* . Ecol Evol 2: 2304–2314. 23139888 10.1002/ece3.346 PMC3488680

[43] Kaltz O, Gandon S, Michalakis Y, Shykoff J (1999) Local maladaptation of the plant pathogen *Microbotryum violaceum* to its host *Silene latifolia*: Evidence from a cross-inoculation experiment. Evolution 53: 395–407. 28565431 10.1111/j.1558-5646.1999.tb03775.x

[44] Giraud T (2004) Patterns of within population dispersion and mating of the fungus *Microbotryum violaceum* parasitising the plant *Silene latifolia* . Heredity 93: 559–565. 15292913 10.1038/sj.hdy.6800554

[45] Gladieux P, Vercken E, Fontaine M, Hood M, Jonot O, et al (2010) Maintenance of fungal pathogen species that are specialized to different hosts: allopatric divergence and introgression through secondary contact. Mol Biol Evol 28: 459–471. 20837605 10.1093/molbev/msq235

[46] Vercken E, Fontaine M, Gladieux P, Hood M, Jonot O, et al (2010) Glacial refugia in pathogens: European genetic structure of anther smut pathogens on *Silene latifolia* and *S. dioica* . PloS Pathog 6: e1001229. 21187901 10.1371/journal.ppat.1001229 PMC3002987

[47] Thomas A, Shykoff J, Jonot O, Giraud T (2003) Mating-type ratio bias in populations of the phytopathogenic fungus *Microbotryum violaceum* from several host species. *Int J Plant Sci* 164: 641–647.

[48] Kaltz O, Shykoff J (2002) Within- and among-population variation in infectivity, latency and spore production in a host-pathogen system. J Evol Biol 15: 850–860.

[49] Queller D, Goodnight K (1989) Estimating relatedness using genetic markers. Evolution 43: 258–275. 28568555 10.1111/j.1558-5646.1989.tb04226.x

[50] Biere A, Honders SC (1996) Host adaptation in the anther smut fungus *Ustilago violacea (Microbotryum violaceum)*: infection success, spore production and alteration of floral traits on two host species and their F1-hybrid. Oecologia 107: 307–320. 28307259 10.1007/BF00328447

[51] West S, Buckling A (2003) Cooperation, virulence and siderophore production in bacterial parasites Proc R Soc Lond B 270: 37–44. 10.1098/rspb.2002.2209 PMC1691207 12590769

[52] Flor HH (1942) Inheritance of pathogenicity in *Melampsoira lini* . Phytopathology 32: 653–669.

[53] St.Clair D (2010) Quantitative disease resistance and quantitative resistance loci in breeding. Ann Rev Phytopat 48: 247–268. 10.1146/annurev-phyto-080508-081904 19400646

[54] Delmotte F, Bucheli E, Shykoff JA (1999) Host and parasite population structure in a natural plant-pathogen system. Heredity 82: 300–308. 10336705 10.1038/sj.hdy.6884850

[55] Bashey F, Young SK, Hawlena H, Lively CM (2012) Spiteful interactions between sympatric natural isolates of *Xenorhabdus bovienii* benefit kin and reduce virulence. J Evol Biol 25: 431–437. 22221661 10.1111/j.1420-9101.2011.02441.x

[56] Kuris AM, Lafferty KD (1994) Community structure: larval trematodes in snail hosts. Ann Rev Phytopat 25: 189–217.

[57] Edwards D, Yu D (2008) Tolerating castration by hiding flowers in plain sight. Behav Ecol Sociobiol 63: 95–102.

[58] Morris W, Doak D (1998) Life history of the long-lived gynodioecious cushion plant *Silene acaulis* (Caryophyllaceae), inferred from size-based population projection matrices. Am J Bot 85: 784–784. 21684962

[59] Dawkins R (1999) The extended phenotype: the long reach of the gene. 2nd Revised edn.: Oxford Paperbacks.

[60] Min K, Lee C, Park H (2012) The lifespan of Korean eunuchs. Current Biol 22: R792–R793. 10.1016/j.cub.2012.06.036 23017989

[61] Neuhaus P, Pelletier N (2001) Mortality in relation to season, age, sex, and reproduction in Columbian ground squirrels (*Spermophilus columbianus*). Can J Zool 79: 465–470.

[62] Thomas F, Teriokhin AT, Renaud F, de Meeûs T, Guegan JF (2001) Human longevity at the cost of reproductive success: evidence from global data. J Evol Biol 13: 409–414.

[63] Lefevre T, Roche B, Poulin R, Hurd H, Renaud F, et al (2008) Exploiting host compensatory responses: the 'must' of manipulation? Trends Parasitol 24: 435–439. 18707919 10.1016/j.pt.2008.06.006

[64] Obeso J (2002) Tansley review: The costs of reproduction in plants. New Phytol 155: 321–348. 10.1046/j.1469-8137.2002.00477.x 33873312

[65] Shykoff JA, Kaltz O (1998) Phenotypic changes in hosts plants diseased by *Microbotryum violaceum*: Parasite manipulation, side-effects, and trade-offs. Int J Plant Sci 159: 236–243.

