# Supplementary material for: Influence of Multiple Infection and Relatedness on Virulence: Disease Dynamics in an Experimental Plant Population and Its Castrating Parasite
Source: PLoS One. 2014 Jun 3;9(6):e98526. doi: 10.1371/journal.pone.0098526 (PMC4043691; doi:10.1371/journal.pone.0098526)
Supplement: Table S2 — Additional analysis of recovery rates. Logistic regression of the recovery rate of Silene latifolia plants in the experimental garden as a function of year, plant sex, relatedness among strains in plants with multiple infections the preceding year and percentage of castrated stems the preceding year; N = 103. This model is presented separately from the one in Table 2 because relatedness within plants in multiple infections cannot be tested in the same model as disease status (multiple versus single infection) because the “single infection” class does not have any relatedness data. The factors plant sex is non-significant when included in the model in Table 2 and it reduces the power to detect the significance of the other factors. (DOCX) [file pone.0098526.s002.docx]

**Table S2:** **Additional analysis of recovery rates.** Logistic regression of the recovery rate of *Silene latifolia* plants in the experimental garden as a function of year, plant sex, relatedness among strains in plants with multiple infections the preceding year and percentage of castrated stems the preceding year; N=103. This model is presented separately from the one in Table 2 because relatedness within plants in multiple infections cannot be tested in the same model as disease status (multiple versus single infection) because the “single infection” class does not have any relatedness data. The factors plant sex is non-significant when included in the model in Table 2 and it reduces the power to detect the significance of the other factors.

|  | **D.f.** | χ **²** | **p** |
| --- | --- | --- | --- |
| **Year** | 1 | 0.066 | 0.7964 |
| **Plant sex** | 1 | 2.988 | 0.0839 |
| **Relatedness among strains in plants with multiple infection the preceding year** | 1 | 0.841 | 0.3592 |
| **Percentage of castrated plants the preceding year** | 1 | 0.190 | 0.6626 |
