# Supplementary material for: Influence of Multiple Infection and Relatedness on Virulence: Disease Dynamics in an Experimental Plant Population and Its Castrating Parasite
Source: PLoS One. 2014 Jun 3;9(6):e98526. doi: 10.1371/journal.pone.0098526 (PMC4043691; doi:10.1371/journal.pone.0098526)
Supplement: Table S3 — Additional analysis of mortality rates. Logistic regression of mortality rate of Silene latifolia plants in the experimental garden as a function of year, plant sex, disease status (multiple vs single infection) the preceding year, and the percentage of diseased stems the preceding year; N = 173. This model is presented separately from the one in Table 3 because the factor “single vs. multiple infections” cannot be tested in the same model as “diseased vs. healthy”. The factor plant sex is non-significant when included in the model in Table 3 and it reduces the power to detect the significance of the other factors. (DOCX) [file pone.0098526.s003.docx]

**Table S3:** **Additional analysis of mortality rates.** Logistic regression of mortality rate of *Silene latifolia* plants in the experimental garden as a function of year, plant sex, disease status (multiple vs single infection) the preceding year, and the percentage of diseased stems the preceding year; N=173. This model is presented separately from the one in Table 3 because the factor “single vs. multiple infections” cannot be tested in the same model as “diseased vs. healthy”. The factor plant sex is non-significant when included in the model in Table 3 and it reduces the power to detect the significance of the other factors.

|  | **D.f.** | χ **²** | **p** |
| --- | --- | --- | --- |
| **Year** | 1 | 23.8589 | <0.00001 |
| **Plant sex** | 1 | 2.4669 | 0.1185 |
| **Relatedness among strains in plants with multiple infection the preceding year** | 1 | 1.3290 | 0.2489 |
| **Percentage of castrated stems the preceding year** | 1 | 0.0004 | 0.9832 |
